# Case report: radiation-induced lumbosacral plexopathy – a very late complication of radiotherapy for cervical cancer

**DOI:** 10.1186/s12883-022-03013-5

**Published:** 2022-12-12

**Authors:** Peter Krkoska, Tomas Kazda, Daniela Vlazna, Blanka Adamova

**Affiliations:** 1 Department of Neurology, Center for Neuromuscular Diseases (Associated National Center in the European Reference Network ERN EURO-NMD), University Hospital Brno, Jihlavska 20, 625 00 Brno, Czech Republic; 2 Faculty of Medicine, Masaryk University, Brno, Czech Republic; 3 Department of Radiation Oncology, Masaryk Memorial Cancer Institute, Brno, Czech Republic; 4 Department of Rehabilitation, University Hospital Brno, Brno, Czech Republic

**Keywords:** Radiation-induced lumbosacral plexopathy, Lumbosacral plexus, Cervical cancer, Radiotherapy, Case report

## Abstract

**Background:**

Lumbosacral plexopathy caused by radiotherapy is a rare but severe consequence of cancer treatment. This condition often leads to varying degrees of sensory and motor impairment. Neurological complications, which are typically permanent, manifest a long period after irradiation.

**Case presentation:**

We describe a case of progressive lower extremity weakness and sensory impairment in a woman who had been effectively treated with radiotherapy for cervical cancer with development 36 years after irradiation. The electrophysiological assessment revealed a subacute bilateral axonal lesion of the lumbosacral plexus. None of the clinical manifestations, serology, cerebrospinal fluid or imaging data discovered an explanation other than radiation-induced lumbosacral plexopathy (RILP).

**Conclusions:**

This case demonstrates that RILP may emerge more than 30 years after the radiotherapy.

## Background

With the increase in long-term survival rates for cancer patients, identifying and managing late treatment-related problems has become a medical and public health concern and an integral part of cancer survivorship care plans [1]. Radiation therapy of the pelvic region is a crucial treatment for various types of cancer that occur in this area, most notably cervical carcinoma [2]. Although peripheral nervous system involvement following radiation therapy is uncommon, one such complication – radiation-induced lumbosacral plexopathy (RILP) – is the most frequent side effect and presents diagnostic and therapeutic challenges [3]. It is a permanent, incurable complication that typically manifests many years after radiation [3]. Previous studies have indicated that neurological issues may manifest up to 30 years following the irradiation [4–8].

We report a case of a cervical cancer patient who had successful oncological treatment but, 36 years after the completion of radiotherapy, developed a severe irradiation-related complication in the form of bilateral RILP.

## Case presentation

In 1979, a 30-year-old woman with no other medical history was admitted to the Masaryk Memorial Cancer Institute in Brno with a diagnosis of cervical squamous cell carcinoma. The initial and most prominent sign was abnormal vaginal bleeding. A gynaecological examination revealed that an exophytic, ulcerated tumour had penetrated the cervix completely but did not extend beyond the vaginal fornices. On the right, the parametrium was not infiltrated; on the left, it had been shortened by an infiltrate in its early stages.

External beam radiotherapy to the pelvic area and radium brachytherapy to the tumour mass were chosen for treatment over six weeks. There was no surgical procedure or chemotherapy.

External beam radiotherapy was administered once a day at a dose of 2 Gy per fraction, five times per week. The total radiation dose to the small pelvis for all 20 fractions was 40 Gy. A betatron with a beam energy of 42 MeV and an anteroposterior field of 20 × 15 cm were used. In addition to this external irradiation, the patient was treated with brachytherapy during the third and fifth weeks of the entire six-week irradiation plan. The source of radiation was radium, during the third week as a vaginal application and during the fifth week as a probe. The total administered dose to the cervix was approximately 70 Gy.

The entire treatment protocol was well tolerated with no short-term adverse effects. A complete remission of the tumour was achieved.

In 2008, 29 years after radiotherapy, late radiation complications began to manifest. The patient was readmitted to the hospital due to severe chronic renal insufficiency caused by tubulointerstitial nephritis. Renal problems were aggravated by bilateral ureterohydronephrosis as a result of severe retroperitoneal fibrosis. This complication resulted in a permanent bilateral nephrostomy. In 2017, the second inpatient care was needed due to severe enterorrhagia. A colonoscopy revealed multiple strictures of the colon, diverticulosis, and histologically verified ischemic colitis.

The nervous system remained symptom-free until 2015, 36 years after the radiotherapy was completed. The patient started to experience mild difficulties with walking. The initial neurological examination revealed bilateral lower extremity weakness with mildly decreased motor function in plantar and dorsal flexion, with the left side being more affected, but no sensory deficit.

**Fig. 1:**
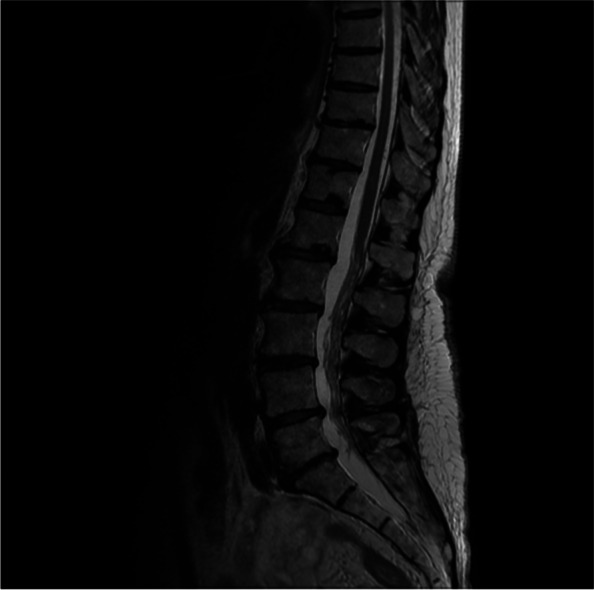
MRI examination of the lumbar spine (T2-weighted scan, sagittal image) 40 years after radiotherapy demonstrates mild degenerative changes but no evidence of spinal stenosis or compression of the lumbosacral nerve roots

In 2017, a decrease in touch sensation at the posterior side of the left lower extremity was described for the first time. The first examination of nerve conduction studies and needle electromyography (NCS/needle EMG) of the lower extremities was misinterpreted as a distal polyneuropathy with bilateral subacute radiculopathy of the nerve roots L5 and S1. The initial magnetic resonance imaging (MRI) of the lumbar spine revealed mild degenerative changes but no spinal stenosis or compression of the lumbosacral nerve roots (Fig. 1). Weakness and hypoesthesia of lower extremities progressively worsened over time and neuropathic pain in the lower extremities appeared, but the control MRI of the lumbar spine remained unchanged.

**Fig. 2:**
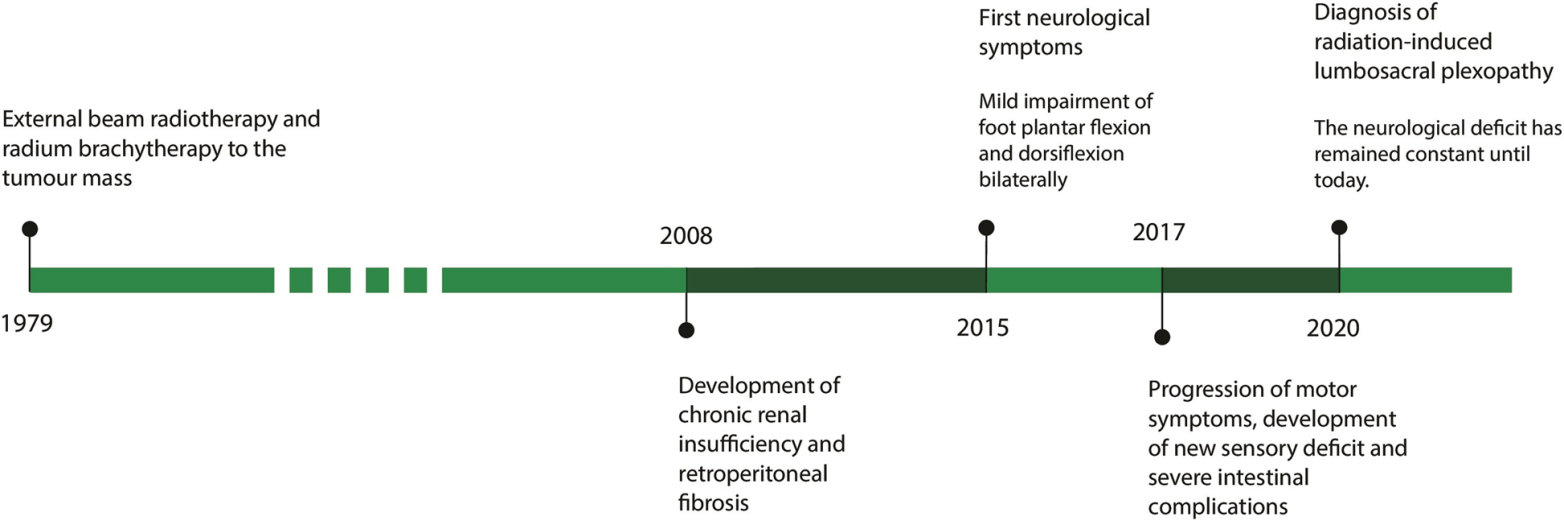
Timeline of the case report

At this point, the patient was referred to a spinal neurologist with expertise in this area (Fig. 2).

The patient underwent a comprehensive neurological evaluation. Manual muscle testing showed bilateral weakness of the lower extremities, accented on the left side. Table 1 shows actual muscle strength of particular muscle groups. Deep tendon reflexes (patellar and Achilles reflexes) were absent bilaterally. A standardised bedside sensory testing of the lower extremities was performed. We used the Neuropen to test tactile sense (10 g monofilament) and sharp pain (tip), the Tip Therm to test temperature sense and the tuning fork to test vibration sense. A sensory examination revealed hypoesthesia for all examined modalities of sensitivity on the calf bilaterally with left-side plantar anaesthesia.

**Table 1:** The results of the muscle test of the patient’s lower extremities using the Medical Research Council scale – a standard for assessing muscle strength in the range of Grade 5 (normal) to Grade 0 (no visible contraction) [9]

*Leg movement*	*Right side*	*Left side*
**Hip abduction**	3	3
**Hip adduction**	4	3
**Hip flexion**	4	3
**Hip extension**	3	2
**Knee flexion**	3	2
**Knee extension**	4	4
**Foot dorsiflexion**	4	3
**Foot plantar flexion**	3	1
**Eversion of the foot**	4	3
**Inversion of the foot**	3	1

The second examination of lower extremity NCS/needle EMG was performed. The examination revealed markedly reduced compound muscle action potential amplitudes of tibial and peroneal nerves on both sides with normal nerve conduction velocities. Sensory nerve action potentials of both sural and superficial peroneal nerves were unelicitable. Needle EMG showed fibrillation potentials, positive sharp waves and complex repetitive discharges together with the enlargement of motor unit potential (MUP) amplitude and duration, their polyphasic shape, and reduced MUP recruitment in all lumbosacral plexus muscles bilaterally. These findings were more pronounced on the left side and in the distribution of the sacral plexus. In the upper extremities, motor and sensory nerve conduction studies of median, ulnar and radial nerves, as well as needle EMG of distal and proximal muscles were normal. The NCS/EMG findings thus supported bilateral subacute axonal lesion of the lumbosacral plexus with dominant sacral plexus impairment, mainly on the left side, which fully corresponded with clinical findings of muscle weakness, which was more pronounced in the sacral plexus distribution with left-sided predominance.

**Fig. 3:**
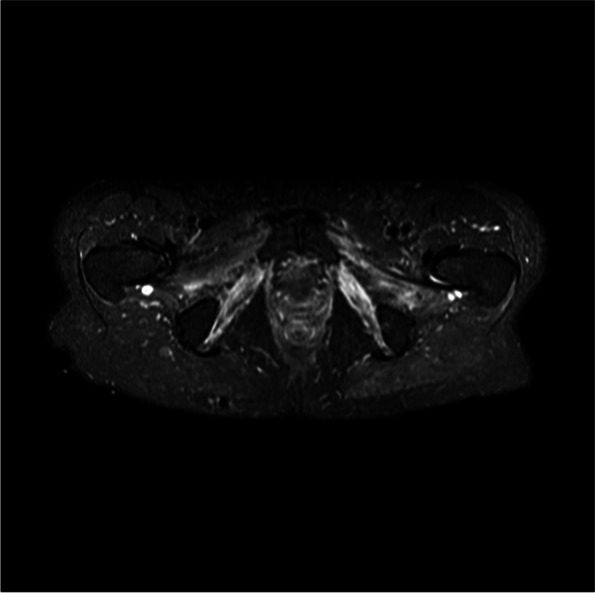
MRI examination of the pelvis (the axial STIR image) 42 years after radiotherapy demonstrates a high signal within the pelvic muscles bilaterally, indicating an acute-to-subacute phase of muscle denervation

After that, an MRI of the pelvis, lumbosacral plexus and spine was performed. There was no evidence of tumour recurrence or metastasis, no pathological enlargement or gadolinium enhancement of the lumbosacral plexus and nerve roots. On the other hand, signs of post-radiation tissue damage were present in multiple locations – retroperitoneal fibrosis, renal atrophy, and colon and rectum wall thickening. Additionally, as seen in Fig. 3, the MRI demonstrated a high signal within the pelvic muscles bilaterally, with left-sided dominance (mostly affected piriformis, external and internal obturator, and pectineus muscles). In accordance with electrophysiological findings, MRI reveals an acute to subacute phase of muscle denervation. Biochemical and haematological analysis revealed no abnormalities apart from signs of chronic renal insufficiency. All other infectious causes (HIV, Lyme disease, herpes simplex and varicella-zoster virus), vasculitis diabetes mellitus were ruled out. Immunofixation for monoclonal proteins, and a panel of paraneoplastic antibodies were also negative. Cerebrospinal fluid testing showed no evidence of nervous system inflammation (protein 0,31 g/L; glucose 0,31 g/L; mononuclears 1,7 × 10^6^/L; polymorphonuclears 0 × 10^6^/L). The diagnosis of radiation-induced bilateral lumbosacral plexopathy was established by the exclusion of other conditions and by correlations between clinical signs and electrophysiological findings.

Due to severe renal insufficiency (creatinine clearance 18.6 ml/min), symptomatic treatment for the neuropathic leg pain was limited. We chose a lower dose of pregabalin (25 mg twice a day), which had a beneficial effect on the neuropathic pain but resulted in lower extremity oedema. A dose reduction to 25 mg every other day was necessary to achieve remission of the oedema while maintaining an effect on the neuropathic pain. The Numeric Rating Scale for Pain (NRS, scale 0–10) was 6 prior to pregabalin therapy and 1 during pregabalin administration.

Due to the restrictions imposed by the Covid-19 pandemic, an individualised home-based rehabilitation programme was used. It consisted of exercises which focused on strengthening and stretching lower extremity muscles. The patient was educated by a physiotherapist on how to perform the task correctly. After a month, there was a check-up visit to validate whether the exercise was being performed accurately. The proposed frequency of training was once a day lasting 15 to 20 min. This programme helped the patient to maintain mobility in her lower extremities. Muscle tests within 2 years remained unchanged as well as sensory impairment. According to administration of the SF-36 questionnaire, the quality of life improved after 1 year of rehabilitation and treatment for neuropathic pain, and remained relatively stable after 2 years, as shown in Table 2.

**Table 2:** The results of the Short Form Health Survey (SF-36)

Health domain	Year 2020	Year 2021	Year 2022
Physical functioning	5%	10%	10%
Role limitations due to physical health	0%	0%	0%
Role limitations due to emotional problems	0%	0%	0%
Energy/fatigue	50%	50%	50%
Emotional well-being	48%	52%	56%
Social functioning	25%	37.5%	37.5%
Pain	22.5%	45%	45%
General health	20%	20%	20%
Health change	0%	75%	50%

## Discussion

We described a cervical cancer patient who received successful radiation treatment but developed a substantial radiotherapy-related long-term complication—RILP. The most remarkable aspect of this case is the occurrence of the RILP 36 years after the therapeutic radiotherapy. To the best of our knowledge, this is the longest time between irradiation and the development of RILP described in the literature.

This example demonstrates that patients presenting with lower extremity weakness, hypoesthesia, or pain more than 30 years after radiation therapy delivered to the pelvis should be evaluated for radiation-induced plexopathy.

Radiation-induced plexopathy is a rare consequence of cancer therapy that may be difficult to diagnose and cure. According to the literature, it is characterised by a dormant interval between radiation exposure and the onset of symptoms [10]. Several cases of delayed manifestation of radiation damage to the lumbosacral plexus have been described in the literature [2, 4, 6, 7, 11].

Numerous risk factors associated with radiotherapy have been identified: large total dose (> 50 Gy to plexus), large dose per fraction (2.5 Gy), heterogeneous high-dose distribution, hot spot high dose, and using an intracavitary radium source [1, 3, 12]. Low-energy devices operated in the 1970s used a close source-to-skin distance, alternating treated fields with steep dosage gradients inside the body [1].

Even though our case did not exceed the dose constraints to the plexus, as the total dose of external beam radiotherapy to the pelvis was 40 Gy, numerous risk factors were involved, including the use of an older generation radiation source (betatron) and the intracavitary radium source.

Radiation-induced adverse effects on the nerve cells may be categorised into early and late stages. Early effects manifest as bioelectrical abnormalities, enzyme modifications, aberrant microtubule assembly, and altered vascular permeability during irradiation. The late effects manifest anytime between one year and decades after irradiation and may be divided into two phases. The first phase is characterised by alterations in the electrophysiology and histochemistry of neurons and glial cells; the second phase is characterised by fibrosis of the nerve-supporting tissue [11, 13]. Recent studies also suggest that compression of nerves produced by severe indirect radiation-induced fibrosis is another essential factor [3]. Thus, the pathogenesis of RILP has not been fully explained; it is a kind of delayed local nerve tissue damage that is partially caused by early microvascular injury followed by radiation-induced fibrosis [1]. In our case, retroperitoneal fibrosis was also present. Its contribution to RILP could be questioned, but it clearly resulted in complications other than neurological, such as uretrohydronephrosis.

The onset of RILP neurological symptoms is typically gradual, with the majority of nerve damage being motor-related. The initial symptoms of RILP are diminished muscular strength. Sensory impairments together with neuropathic pain are typically discovered later. Symptoms are usually bilateral and asymmetric, with initial unilateral damage [1, 3, 14]. Our case fits this description; the disease manifested initially as bilateral and asymmetric lower extremity weakness, followed by sensory impairment and neuropathic pain.

The diagnosis of RILP is established by the exclusion of other conditions. Differential diagnoses include particularly metastasis and local tumour growth that result in direct compression or infiltration of the lumbosacral plexus or lumbosacral roots, neuroinfection, connective tissue diseases, systemic vasculitis,polyneuropathy, spondylogenic disorders especially lumbar spinal stenosis with compression of lumbosacral roots. Minimal workup should include laboratory studies, cerebrospinal fluid analysis, NCS/needle EMG, and radiological imaging, preferably MRI, of the pelvis and lumbar spine [15, 16].

The initial examination thus usually includes radiological imaging of the lumbar spine and pelvis, which is crucial to rule out tumour invasion and spondylogenic compression of the lumbosacral roots in the lumbar spinal canal. Although cerebrospinal fluid analysis is critical for ruling out malignant cells or infection, it may detect elevated cerebrospinal fluid protein level in some RILP patients but does not reveal any specific findings [1, 3, 10]. The NCS/needle EMG of the lower extremities is used to determine the location, phase and severity of plexus injury [1].

Three criteria for determining the diagnosis of RILP have been established: 1) there is a history of radiotherapy that includes the plexus, 2) the primary neurologic lesion is contained within the radiotherapy-exposed segments, 3) metastatic and other disorders of the plexus have been ruled out [3]. Our patient met these three criteria. Initially, however, the diagnosis of RILP was not considered because the lower extremity NCS/needle EMG was misinterpreted, and spondylosis of the lumbar spine was overestimated.

In contemporary clinical practice, there is no definitive treatment for RILP. The therapeutic modalities should be aimed at relieving pain, paraesthesia, psychological distress, strengthening the muscles, and preserving the range of motion in joints of the lower extremity. Once the severe weakness is established, it is extremely unlikely to recover regardless of treatment due to severe axonal damage [1, 3, 10, 17].

Neuropathic pain is the main type of pain in RILP patients. Several recommendations and guidelines for neuropathic pain pharmacotherapy have been proposed [18, 19]. In light of these recommendations, tricyclic antidepressants, serotonin-noradrenaline reuptake inhibitor (SNRI) antidepressants, pregabalin, and gabapentin are the most suitable for the treatment of neuropathic pain [18]. However, the treatment of neuropathic pain should be comprehensive, and rehabilitation is also recommended (in the form of a tailored rehabilitation programme and physical therapy), as are neurostimulation techniques (e.g. transcutaneous electrical nerve stimulation, high-frequency repetitive transcranial magnetic stimulation of the motor cortex) [19, 20]. Psychotherapy is an integral part of neuropathic pain treatment (cognitive behavioural therapy and mindfulness), recommended as a second-line therapy, as an add-on to other therapies [19]. Rehabilitation of RILP patients assists not only in pain relief but also in strengthening the muscles and avoiding joint contractures.

In our case, pregabalin was used because of its tolerability, effectiveness in alleviating neuropathic pain, and ability to improve mood states in patients with radiotherapy-related neuropathic pain with great effect [17]. Even though the pregabalin dose was low (25 mg once every two days) due to severe renal insufficiency and lower extremity oedema, the effect on neuropathic pain was excellent because pain intensity as assessed by the NRS was reduced by 83% with pregabalin administration.

Two years after the initial admission to our clinic, the patient’s lower extremity neuropathic pain was well tolerated on pregabalin and the neurological deficit remained stable, so sensory impairment and pareses of the lower extremities remained unchanged. Even though the pareses have not worsened over the last two years and the neuropathic pain has been manageable, there is no expectation that patient’s motor deficit would be completely recovered. However, all used therapeutic modalities have aimed to preserve the patient’s quality of life and ability to participate in activities of daily living.

As a result of the lack of a curative method, the optimal approach is prevention. When radiotherapy to the small pelvis is chosen as the treatment modality, the optimal strategy is to avoid exceeding dose-volume constraints to pertinent at-risk organs by employing state-of-the-art radiotherapy technologies (for example volumetric modulated arc therapy) [21].

## Conclusions

RILP is a rare but severe consequence of radiotherapy to the small pelvis that can manifest a long period after irradiation. The diagnosis requires the exclusion of other causes of involvement of the lumbosacral plexus. There is no definitive treatment for RILP and the therapy should be complex and symptomatic. The optimal approach is prevention by employing state-of-the-art radiotherapy technologies.

## Data Availability

All data generated or analysed during this study are included in this published article and its supplementary information files.

## Abbreviations

HIV: Human immunodeficiency virus
Needle EMG: Needle electromyography
NCS: Nerve conduction studies
RILP: Radiation-induced lumbosacral plexopathy
SF-36: 36-Item Short Form Survey
